# Data on graphical representation (CGR and FCGR) of bacterial and archaeal species from two Soda Lakes

**DOI:** 10.1016/j.dib.2017.03.017

**Published:** 2017-03-16

**Authors:** Bhagwan N. Rekadwad, Chandrahasya N. Khobragade

**Affiliations:** School of Life Sciences, Swami Ramanand Teerth Marathwada University, Nanded 431606, MS, India

**Keywords:** Chilka Lake, ENDMEMO, Halophiles, Sambhar Lake, 16S rRNA

## Abstract

In this paper, we presented the datasets generated using Chose Game representation (CGR) and Choase Game Representation of Frequencies (FCGR) of bacterial and archaeal 16S rRNA sequences. The data in the form of graphical representations was yielded with the help of ENDMEMO tool. The computational representation of these data datasets is useful for studies and interpretation of microbial sequences. Based on a technique from chaotic dynamics, the method produces a picture of any gene (DNA and RNA) sequence which displays both local and global patterns. Eukaryotes and prokaryotes can be identified merely based on their generated visual representation/DNA structures.

**Specifications Table**

**Table:** 

Subject area	*Biology*
More specific subject area	*Microbiology; Bioinformatics*
Type of data	*Table, figure*
How data was acquired	*Bioinformatics tools*
Data format	*Raw, analyzed*
Experimental factors	*Standard and default*
Experimental features	*Graphical representations*
Data source location	*School of Life Sciences, SRTM University, Nanded*
Data accessibility	*Data is available with this article*

**Value of the data**

• Data generated in this study permits the representation and investigation of patterns in any type of sequences which visually revealed previously unknown pattern.
• The generated graphical data by means of sequences using a new tool derived from the "chaotic dynamical systems" which allowed the depiction of frequencies of oligonucleotides in the form of images.
• Data on CGR and FCGR are the main factors explaining the variability observed among sequences. The distance between images helpful for measurement of phylogenetic proximity.

## 1 Data

This paper describes data on 16S rRNA sequence of bacterial and archaeal species isolated from Soda Lakes such as Sambhar Lake and Chilka Lake (India). The data generated in the form of graphical representations contains information on their oligonucleotides distribution and numbers.

## 2 Experimental design, materials and methods

115 bacterial and archaeal 16S rRNA sequences (both short and long) were obtained in FASTA format from NCBI repository (Table 1). These sequences of bacteria and archaea were used for graphical representations. The generated graphical representations in the form of Chaose Game Representations (Fig. 1) and Chose Game Representations of Frequencies (Fig. 2) obtained in the form of visual images [1], [2]. Graphical representations of oligonucleotides in the form of CGR and FCGR pictorial representations were created using ENDMEMO tool [3], [4] for studies on primary sequence organization and representation of oligonucleotides frequency in the given sequence.

## Figures and Tables

**Table 1:** Bacterial and archaeal species isolated from Soda Lakes.

**Accession number**	**Species/Strain**
AF472595	Sambhar Salt Lake archaeon HA1
AF472596	Sambhar Salt Lake archaeon HA6
AJ889020	*Marichromatium chilcum*
EU669822	Haloalkaliphilic bacterium EMB4
FJ984520	*Marinobacter alkaliphilus* strain NBSL05
FJ984521	*Marinobacter alkaliphilus* strain NBSL06
FJ984522	*Marinobacter hydrocarbonoclasticus* strain NBSL04
FJ984523	*Halomonas* sp. NBSL08
FJ984524	*Marinobacter alkaliphilus* strain NBSL03
FJ984525	*Halomonas* sp. NBSL10
FJ984526	*Halomonas* sp. NBSL14
FJ984527	*Tsukamurella* sp. NBSL21
FJ984528	*Ochrobactrum haemophilum* strain NBSL23
FJ984529	*Bacillus horikoshii* strain NBSL26
FJ984530	*Bacillus horikoshii* strain NBSL27
FJ984531	*Micrococcus luteus* strain NBSL29
FJ984532	*Halomonas* sp. NBSL09
FJ984533	*Micrococcus luteus* strain NBSL28
GQ227415	*Methylobacterium radiotolerans* strain NBCS1
GQ281064	*Hyphomicrobium facile* strain NBCS26
GQ281065	*Methylobacterium zatmanii* strain NBCS25
GQ281066	*Hyphomicrobium facile* strain NBCS23
GQ281070	*Mycobacterium brisbanense* strain NBCS10
GQ281073	*Pseudomonas* sp. NBCS06
GQ281075	*Hyphomicrobium* facile strain NBCS7
GQ354269	*Methylobacterium radiotolerans* strain NBCS3
GQ411500	*Methylobacterium extorquens* strain NBCS16
GQ411502	*Methylobacterium* sp. NBCS19
GQ411503	*Methylobacterium radiotolerans* strain NBCS21
GQ411505	*Methylobacterium* sp. NBCS20
GQ411539	Uncultured *Streptosporangium* sp. clone 62
GQ411540	Uncultured *Streptosporangium* sp. clone 61
GQ411541	Uncultured *Streptosporangium* sp. clone 64
GQ411542	Uncultured *Streptosporangium* sp. clone 63
JF343124	*Staphylococcus* sp. IARI-CS-2
JF343125	*Acinetobacter johnsonii* strain IARI-CS-7
JF343126	*Acinetobacter venetianus* strain IARI-CS-13
JF343127	*Acinetobacter* sp. IARI-CS-15
JF343128	*Acinetobacter* sp. IARI-CS-17
JF343129	*Micrococcus luteus* strain IARI-CS-18
JF343130	*Agromyces* sp. IARI-CS-28
JF343132	*Micrococcus indicus* strain IARI-CS-31
JF343133	*Staphylococcus haemolyticus* strain IARI-CS-32
JF343139	*Bacillus mycoides* strain IARI-CS-41
JF343140	*Bacillus altitudinis* strain IARI-CS-43
JF343144	*Acinetobacter venetianus* strain IARI-CS-50
JF343145	*Acinetobacter venetianus* strain IARI-CS-51
JF343152	*Staphylococcus arlettae* strain IARI-CS-60
JF343153	*Pseudomonas stutzeri* strain IARI-CS-62
JF343157	*Exiguobacterium* sp. IARI-CS-68
JF343158	*Exiguobacterium indicum* strain IARI-CS-69
JF343162	*Micrococcus yunnanensis* strain IARI-CS-16
JF343163	*Sphingomonas melonis* strain IARI-CW-25
JF343165	*Stenotrophomonas* sp. IARI-CW-51
JF343167	*Staphylococcus equorum* strain IARI-CW-11
JF343170	*Pseudomonas aeruginosa* strain IARI-CW-30
JN411473	*Stenotrophomonas* sp. IARI-CW-52
JN411475	*Stenotrophomonas* sp. IARI-CW-55
JQ328187	*Natronococcus* sp. SLA-60
JQ328188	*Natronococcus occultus* strain SLA-2
JQ328189	*Natronococcus* sp. SLA-3
JX428952	*Halobacillus* sp. IARI-ABCL-1
JX428953	*Nesterenkonia halophila* strain IARI-ABCL-4
JX428954	*Halococcus* sp. IARI-ABCL-7
JX428955	*Brachybacterium* sp. IARI-ABCL-8
JX428956	*Pontibacillus* sp. IARI-ABCL-9
JX428957	*Virgibacillus halodenitrificans* strain IARI-ABK-2
JX428958	*Marinococcus halophilus* strain IARI-ABK-3
JX428959	*Haladaptatus paucihalophilus* strain IARI-ABK-4
KC434452	*Halomonas* sp. SSL5
KC434453	*Halomonas venusta* strain SSL6
KC434454	*Oceanobacillus* sp. SSL7
KC434455	*Natronococcus xinjiangense* strain SLA61
KC434456	*Halomonas pantelleriensis* strain SSL8
KC434457	*Natronorubrum thiooxidans* strain SLA62
KC440854	*Bacillus* sp. SSL1
KC440855	*Bacillus cereus* strain SSL2
KC696560	*Natronococcus occultus* strain SLA64
KC696561	*Natronococcus* sp. SSL9
KC820814	*Natronococcus jeotgali* strain SLA63
KC924847	*Euhalothece* sp. SLVH01
KC934935	*Nesterenkonia* sp. SSL10
KC934936	*Halomonas* sp. SSL11
KC934937	*Oceanobacillus iheyensis* strain SSL12
KC934938	*Halomonas alkaliphila* strain SSL13
KC934939	*Halomonas* sp. SSL14
KC934940	*Halomonas pantelleriensis* strain SSL15
KC934941	*Staphylococcus* sp. SSL16
KF288960	*Halomonas* sp. SSL3
KF288961	*Halomonas pantelleriensis* strain SSL4
KU179507	*Microbacterium* sp. ANSKSlab01
KU518891	*Paenibacillus dendritiformis* strain ANSKLAB02
KU529483	*Bacillus tequilensis* strain ANSKLAB04
LT161878	*Paenibacillus* sp. SMB1
LT161879	*Halomonas* sp. SMB2
LT161880	*Halomonas* sp. SMB3
LT161881	*Bacillus* sp. SMB4
LT161882	*Bacillus* sp. SMB5
LT161883	*Bacillus* sp. SMB6
LT161884	*Halomonas* sp. SMB7
LT222351	*Halomonas* sp. SMB8
LT599833	*Exiguobacterium* sp. SMB10
NZ_MASN01000022	*Natrialba* sp. SSL1 ctg29
NZ_MASN01000031	*Natrialba* sp. SSL1 ctg37
NZ_MASN01000032	*Natrialba* sp. SSL1 ctg38
NZ_MASN01000033	*Natrialba* sp. SSL1 ctg39
NZ_MASN01000042	*Natrialba* sp. SSL1 ctg47
NZ_MASN01000044	*Natrialba* sp. SSL1 ctg49
NZ_MASN01000045	*Natrialba* sp. SSL1 ctg5
NZ_MASN01000046	*Natrialba* sp. SSL1 ctg50
NZ_MASN01000047	*Natrialba* sp. SSL1 ctg51
NZ_MASN01000049	*Natrialba* sp. SSL1 ctg53
NZ_MASN01000053	*Natrialba* sp. SSL1 ctg57
NZ_MASN01000054	*Natrialba* sp. SSL1 ctg58
NZ_MASN01000055	*Natrialba* sp. SSL1 ctg59
NZ_MASN01000057	*Natrialba* sp. SSL1 ctg60
NZ_MASN01000058	*Natrialba* sp. SSL1 ctg61

## Footnotes

Appendix A Supplementary data associated with this article can be found in the online version at doi:10.1016/j.dib.2017.03.017.
