# Supplementary material for: Data on graphical representation (CGR and FCGR) of bacterial and archaeal species from two Soda Lakes
Source: Data Brief. 2017 Mar 16;12:72–6. doi: 10.1016/j.dib.2017.03.017 (PMC5376247; doi:10.1016/j.dib.2017.03.017)
Supplement: Supplementary file 2 — Supplementary material. [file mmc2.docx]

| 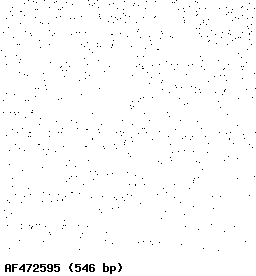 | 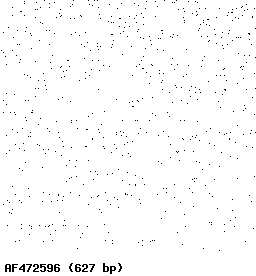 | 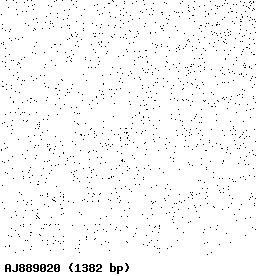 |
| --- | --- | --- |
| 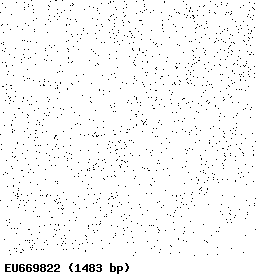 | 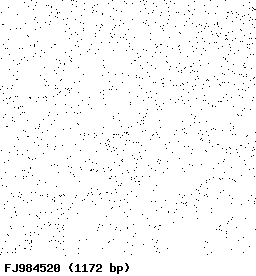 | 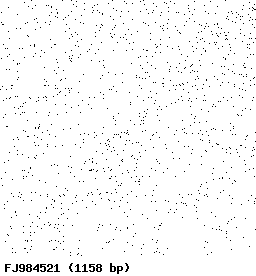 |
| 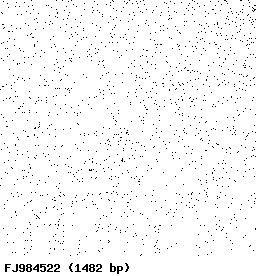 | 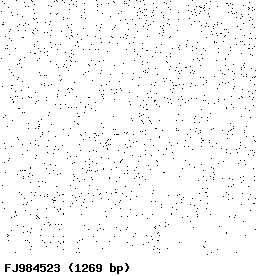 | 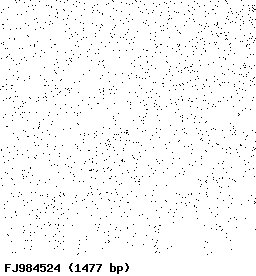 |
| 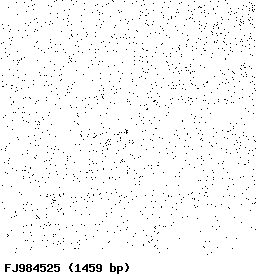 | 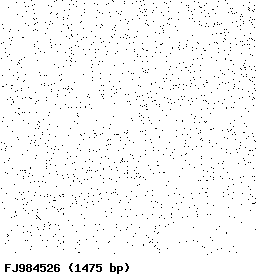 | 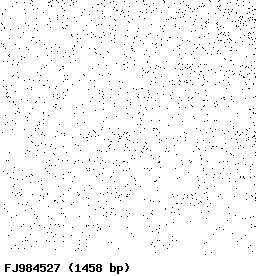 |
| 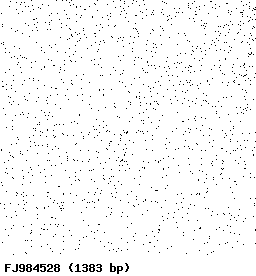 | 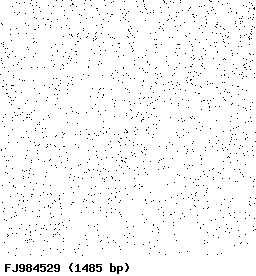 | 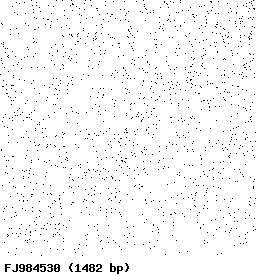 |
| 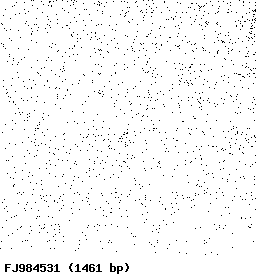 | 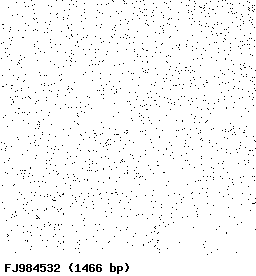 | 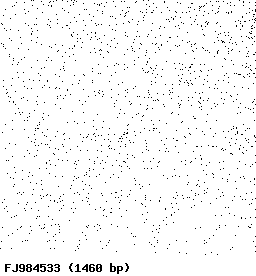 |
| 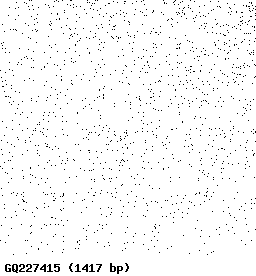 | 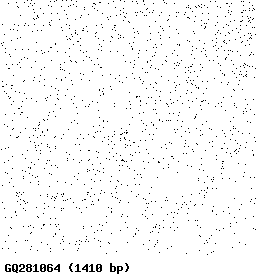 | 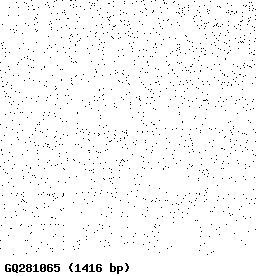 |
| 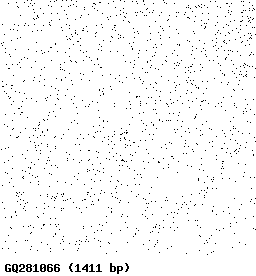 | 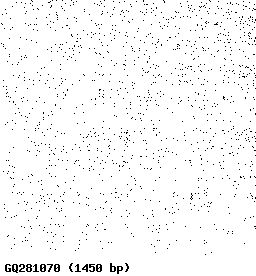 | 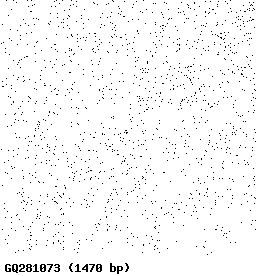 |
| 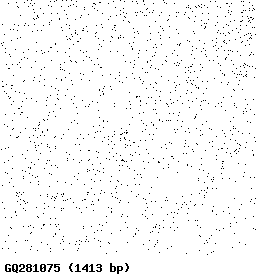 | 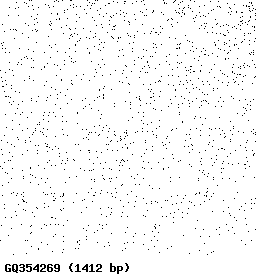 | 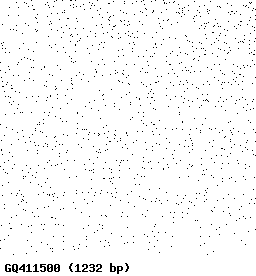 |
| 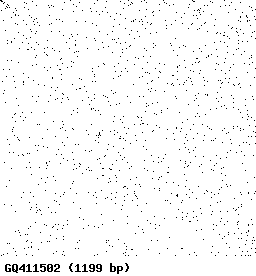 | 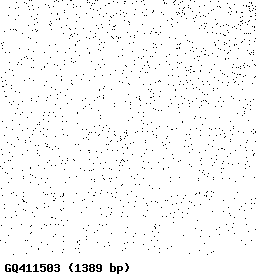 | 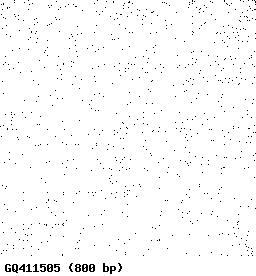 |
| 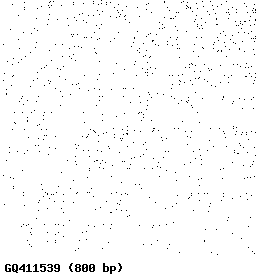 | 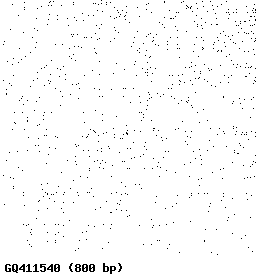 | 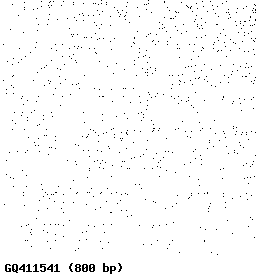 |
| 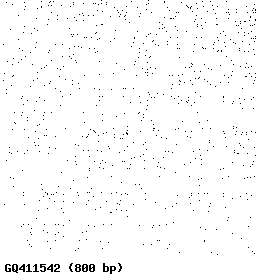 | 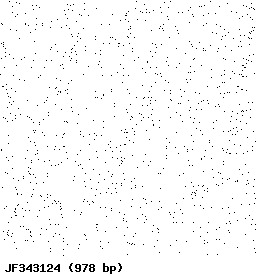 | 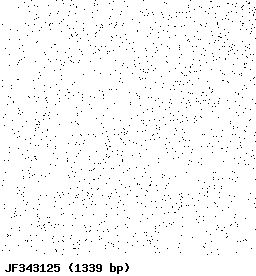 |
| 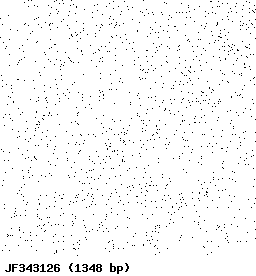 | 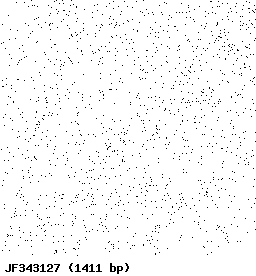 | 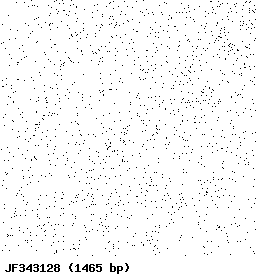 |
| 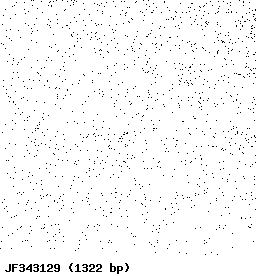 | 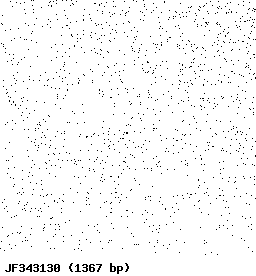 | 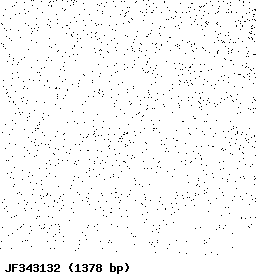 |
| 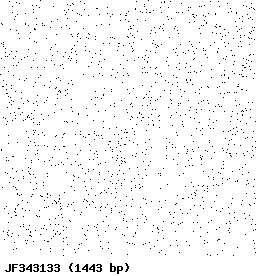 | 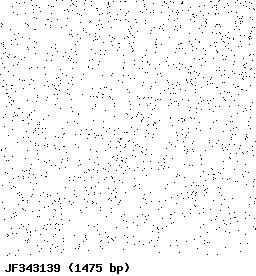 | 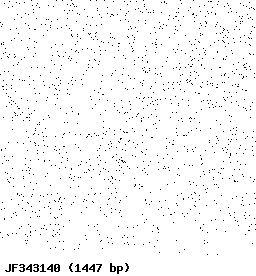 |
| 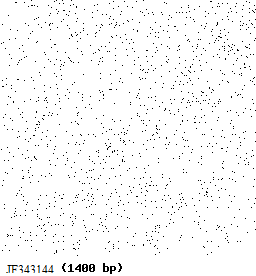 | 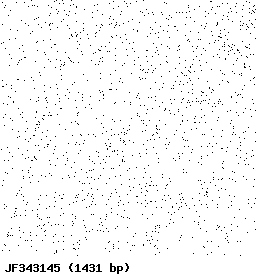 | 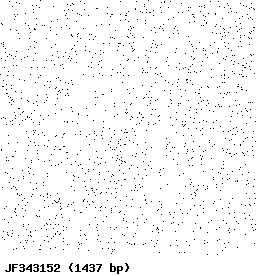 |
| 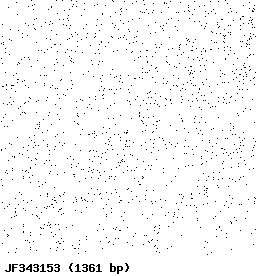 | 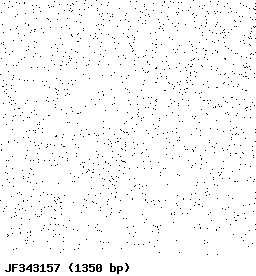 | 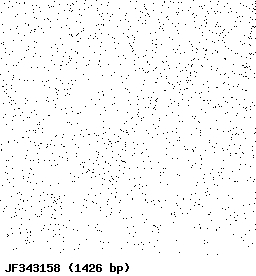 |
| 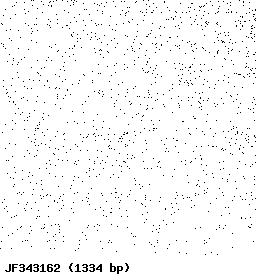 | 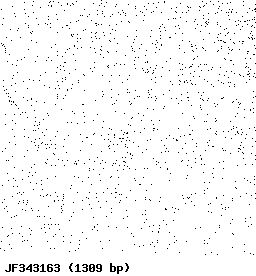 | 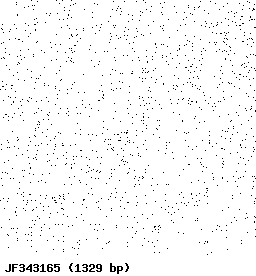 |
| 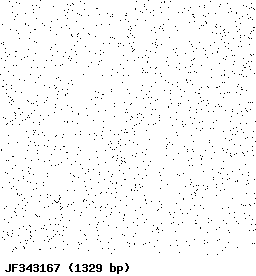 | 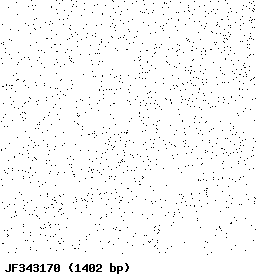 | 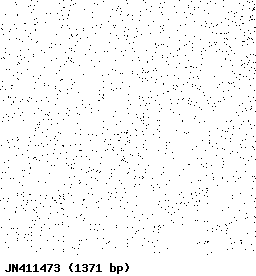 |
| 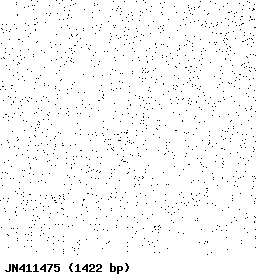 | 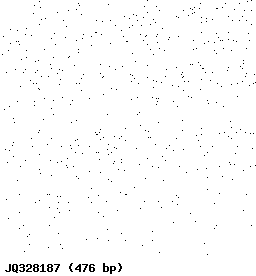 | 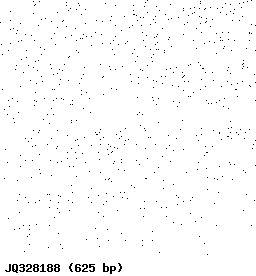 |
| 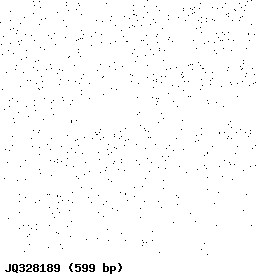 | 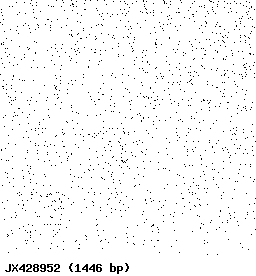 | 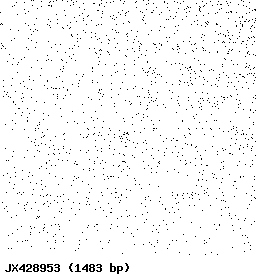 |
| 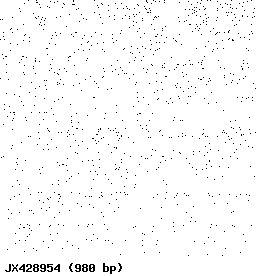 | 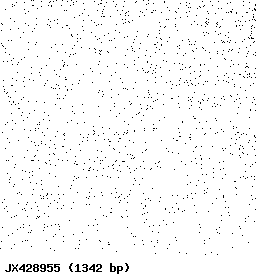 | 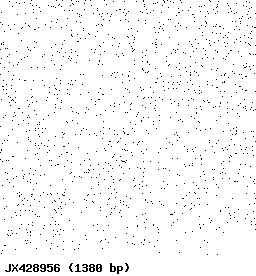 |
| 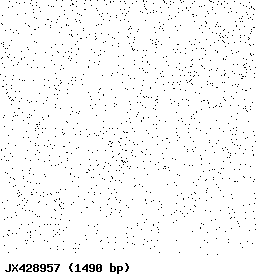 | 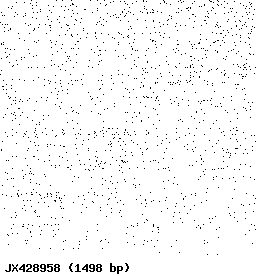 | 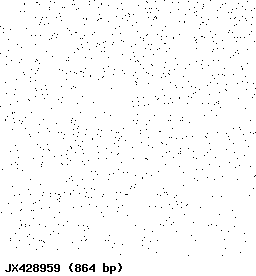 |
| 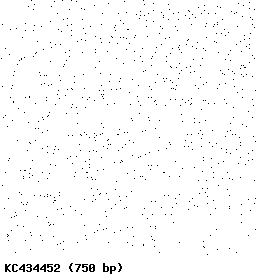 | 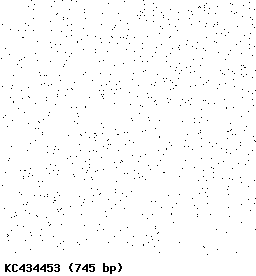 | 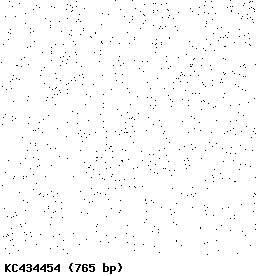 |
| 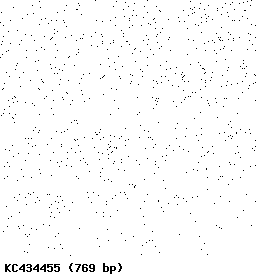 | 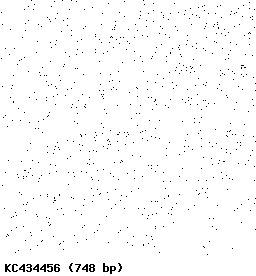 | 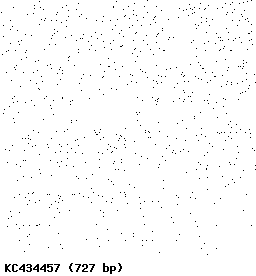 |
| 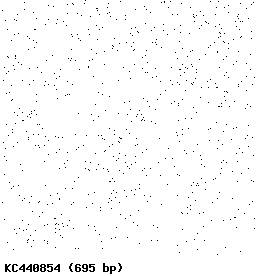 | 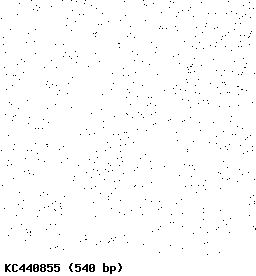 | 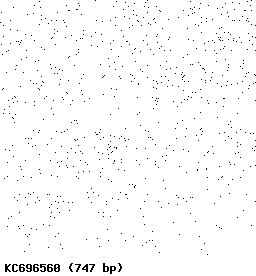 |
| 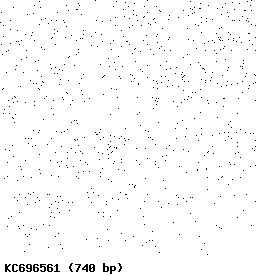 | 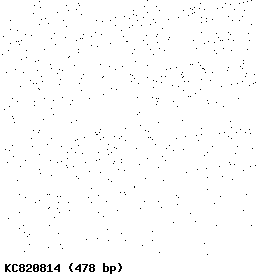 | 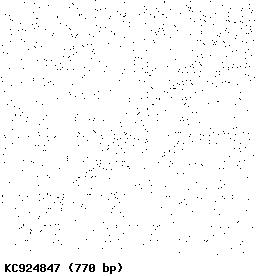 |
| 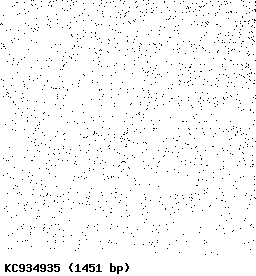 | 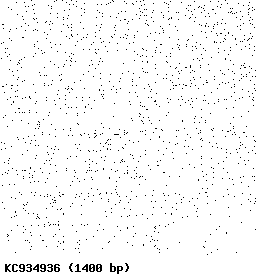 | 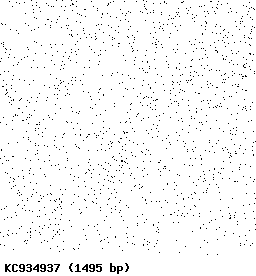 |
| 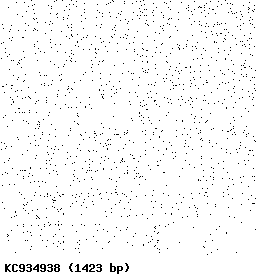 | 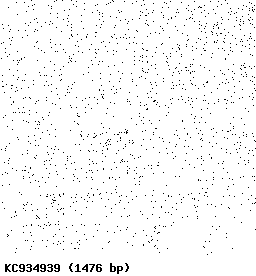 | 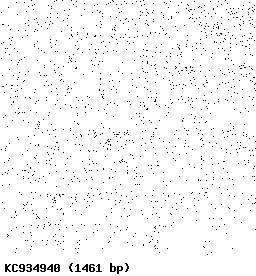 |
| 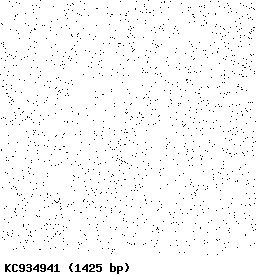 | 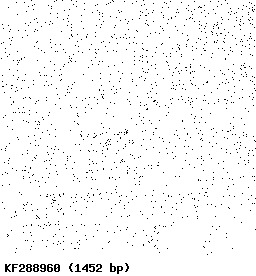 | 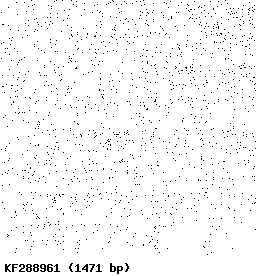 |
| 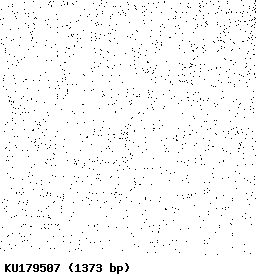 | 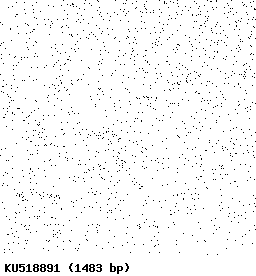 | 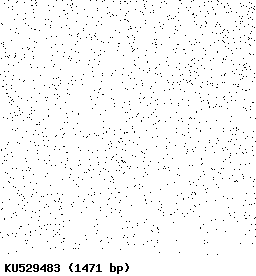 |
| 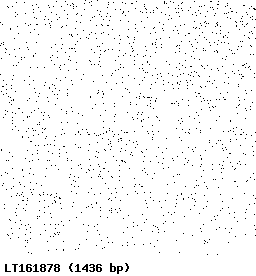 | 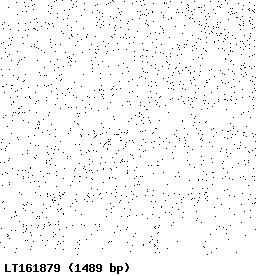 | 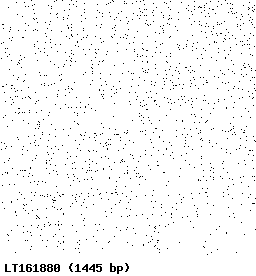 |
| 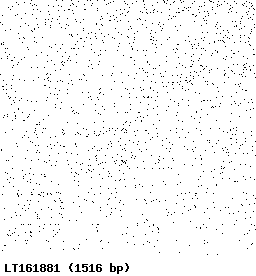 | 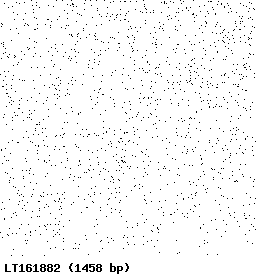 | 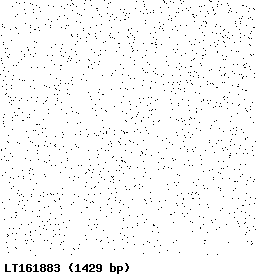 |
| 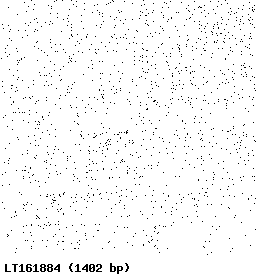 |  |  |
|  |  |  |
|  |  |  |
|  |  |  |
|  |  |  |
|  |  |  |

**Fig.1** Chaose Game Representation of Frequencies (FCGR) of Soda Lake isolates

**Fig.2** Chaose Game Representation of Frequencies (FCGR) of Soda Lake isolates
